# Peripheral Osteoma of the Mandibular Condyle—Case Series

**DOI:** 10.3390/dj10100182

**Published:** 2022-09-29

**Authors:** Ioannis Tilaveridis, Theodora Katopodi, Panagiotis Karakostas, Gregory Venetis, Ioannis Dimitrakopoulos, Stavros Tilaveridis, Sofia Tilaveridou, Katerina Zarampouka

**Affiliations:** 1 Department of Oral & Maxillofacial Surgery, School of Dentistry, Aristotle University of Thessaloniki, 54636 Thessaloniki, Greece; 2 Department of Laboratory of Medical Biology-Genetics, School of Medicine, Aristotle University of Thessaloniki, 54636 Thessaloniki, Greece; 3 Department of Preventive Dentistry, Periodontology and Implant Biology, School of Dentistry, Aristotle University of Thessaloniki, 54636 Thessaloniki, Greece; 4 Department of Pathology, Medical School, Aristotle University of Thessaloniki, 54636 Thessaloniki, Greece

**Keywords:** osteoma, peripheral, mandible, condyle

## Abstract

The purpose of this article is to present four new cases of peripheral osteoma of the mandibular condyle and the literature review. A retrospective study of files from our Department of Oral and Maxillofacial Surgery over the last 6 years revealed four cases of peripheral osteomas located in the area of the mandibular condyle. Diagnostic procedure included clinical, radiographic, and histologic criteria. Gardner’s syndrome was excluded from patient history and clinical evaluation. One patient had only an aesthetic disturbance, with facial swelling, and the other three patients presented disturbances of the mandibular function, including deviation during mouth opening along with malocclusion. Three of the patients were male and one was female; all were of middle age (45–65 years old). The proposed surgical treatment was accepted by half of the patients, while the remaining half declined the operation after a confirmation of the diagnosis. Peripheral osteomas of the maxillofacial region are uncommon, and some cases with multiple osteomas are related to Gardner’s syndrome. An osteoma of the mandibular condyle is very rare and surgical treatment is challenging for the surgeon with regards to the approach selection and the related complications. In the two cases that accepted the proposed surgical treatment, no recurrence and no complication was observed.

## 1. Introduction

Osteoma is a benign osteogenic tumor arising either from the endosteum or the periosteum with the respective lesions, and accordingly, these lesions are categorized as central or peripheral. A third category of osteomas includes those that are located in the soft tissue, which are characterized as extra-skeletal [1,2]. Peripheral osteomas, or exostoses, arise from the periosteum, and clinically, they are well-circumscribed, slow-growing, and benign in nature bony protuberances, usually in young adults [1]. Although the exact pathogenesis of osteomas is still unclear, traumatic, developmental, congenital, inflammatory, and endocrine causes have been considered as possible etiologic factors [3,4].

Osteomas are more commonly found into the paranasal sinuses, especially in the frontal sinus, and only rarely are they encountered in the mandible. In some patients, they are related to Gardner’s syndrome. This syndrome includes not only multiple unerupted teeth but also multiple osteomas of the mandible, supernumerary teeth, and odontomas located in the facial bones. Among these cases, most common sites are the mandibular angle and the inferior rim of the mandibular body [5]. Peripheral osteomas of the mandibular condyle are extremely rare, and their surgical treatment, when they are in this region, is challenging for the surgeon. In our paper, we present four new cases of peripheral osteomas at the mandibular condyle, along with the proposed treatment, and a review of the literature.

## 2. Case Series

### 2.1. Case 1

**Figure 1:**
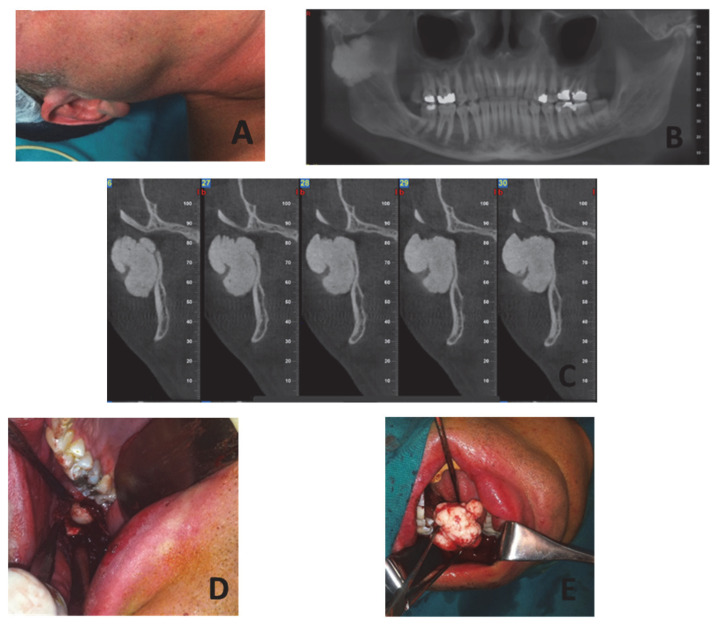

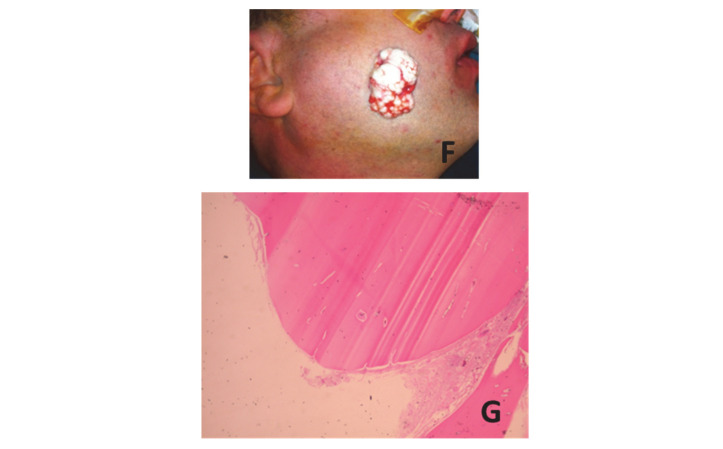
(**A**) Extra-oral swelling at the right pre-auricular area; (**B**) Panoramic X-ray showing a radiopaque well-circumscribed lesion at the area of the right mandibular condyle; (**C**) Coronal view of CT showing the lesion in relation to the ramus; (**D**) Intra-oral exposure of the osteoma; (**E**) Osteoma after detachment from the mandibular condyle; (**F**) Complete removal of the osteoma; (**G**) Dense compact bone with well-defined borders H & E X25.

A 45-year-old male was presented to our office with an extra-oral swelling located at the right pre-auricular region, dating back over a year. The swelling was painless, compact in palpation, and had slowly increased in size over the last months (Figure 1A). His history was free and the patient referred no previous trauma, infection, or any other irritating factor at the affected area. The mouth opening was normal, without pain during chewing. The neck palpation was negative for lymphadenitis of the regional lymph nodes. A panoramic X-ray revealed a compact radio-opaque lesion on the external surface of the right mandibular condyle (Figure 1B). A CT-imaging revealed a 4 × 3 × 1.5 cm^3^ well-defined, radio-opaque lesion (Figure 1C). Imaging findings, along with history and clinical findings, raised the suspicion of a benign bone tumor of the right mandibular condyle. Differential diagnosis included osteoma, osteochondroma, or chondroma. A diagnosis of Gardner’s syndrome was excluded due to absence of multiple osteomas, especially in the facial bones, abnormalities of the remaining skeleton, sebaceous cysts, or a history of adenomatous polyps. Moreover, the patient was alerted to keep in contact with a gastroenterologist.

Detailed information of the diagnosis was recorded and patient consent for the surgical removal of the lesion was obtained. The surgical plan included an intra-oral approach in an attempt to remove the osteoma, and to avoid extra-oral incisions which had the related risk of facial nerve damage. In case of a failure to remove the osteoma through the intra-oral route, a standard pre-auricular approach was considered.

The patient was operated on under general nasotracheal anesthesia and an intra-oral incision was performed in the anterior edge of the ramus, similar to the one performed in a sagittal split osteotomy. Obwegeser retractors were used to achieve the best visibility of the surgical field. The osteoma was recognized and completely removed with chisels in one piece (Figure 1D–F). The wound was closed with interrupted sutures and the immediate post-operative course was uneventful, without serious complication, except for the expected extra-oral post-surgical edema and a mild difficulty in mouth opening. A soft diet for 10 days and active jaw opening were applied. The histopathologic report showed that the tumor consisted of dense mature bone with lacuna, and the diagnosis of a peripheral osteoma of the condyle was established (Figure 1G). During follow-up, no symptoms or recurrence were recorded.

### 2.2. Case 2

A 48-year-old male patient was presented to our clinic with a swelling in the right pre-auricular region and facial asymmetry with a disturbance of the occlusion. Symptoms had started 19 months earlier, and during the last eight months, the situation remained relatively stable. The medical history of the patient was non-contributory, and no previous trauma or infection was mentioned.

**Figure 2:**
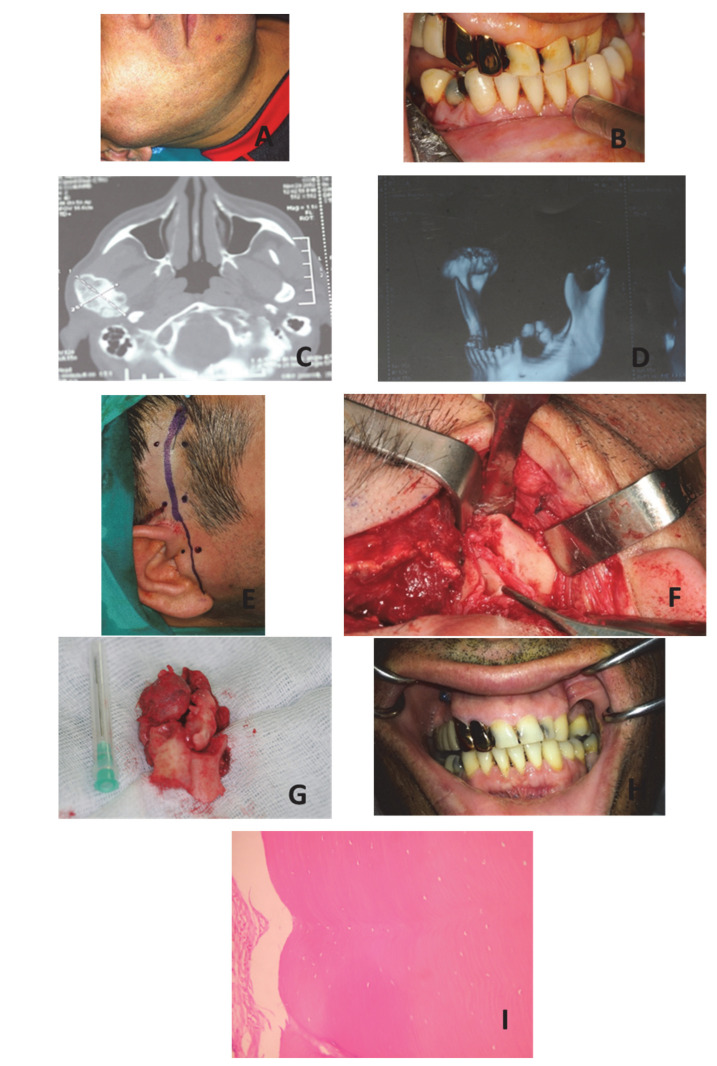
(**A**) Right pre-auricular swelling; (**B**) Posterior open bite on the right and transposition of the mandibular midline on the left; (**C**) Transverse view of CT showing the osteoma of the right mandibular condyle; (**D**) 3D reconstruction of the mandible showing the enlargement of the condyle; (**E**) Pre-auricular incision with temporal extension; (**F**) Exposure of the right mandibular condyle; (**G**) Osteoma after its extirpation; (**H**) The occlusion after removal of the osteoma; (**I**) Compact bone with very sparse arrangement of osteocyte and well-defined borders H & E X200.

A clinical evaluation revealed a hard extraoral swelling in the right condylar area (Figure 2A), and facial asymmetry in the lower third of the face. There was an intense malocclusion with a posterior open bite on the right affected side and a cross-bite on the left (Figure 2B). The mandibular midline was deviated 6 mm to the left. The mouth opening was normal in range with the deviation but without serious temporomandibular disorder, although his main complaint was chewing difficulty.

A panoramic X-ray showed an enlargement of the right mandibular condyle, and a 3D reconstruction CT-imaging revealed a large osteoma of the right mandibular condyle, which was well-circumscribed from the surrounding condylar head (Figure 2C,D). The suspicion of a peripheral osteoma was raised, but the differential diagnosis also included chondroma and osteochondroma. A history and clinical signs or symptoms of Gardner’s syndrome were absent and a gastroenterologist’s consultation excluded Gardner’s symptoms. Written consent for the surgical removal of the lesion was obtained and the operation was performed under general nasotracheal intubation. A standard pre-auricular incision with temporal extension was selected, and the affected area was exposed (Figure 2E–H) with special care to avoid trauma of the facial nerve branches. The osteoma was dissected and removed in pieces, leaving the head of the condyle intact. Recovery was uneventful, without complication from the facial nerve. The jaw opening was restricted for 7 days, and active jaw exercises were recommended. Histopathologic examination confirmed the initial diagnosis of **a** peripheral condylar osteoma consisting mainly of dense bone (Figure 2I). The patient was followed-up and remains with a normal occlusion and without clinical symptoms.

### 2.3. Case 3

**Figure 3:**
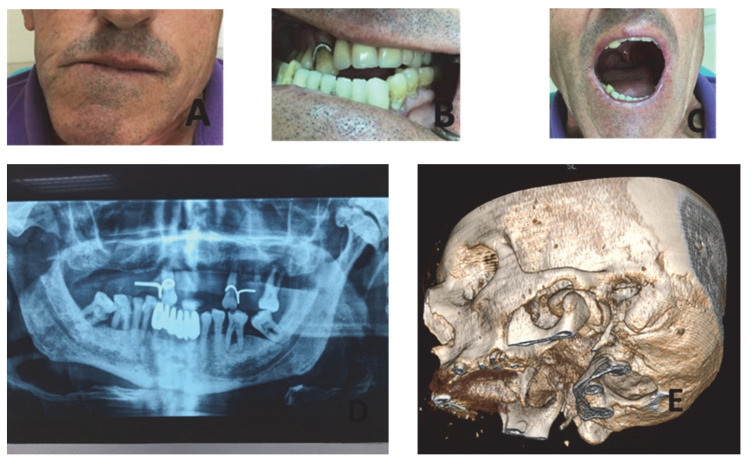

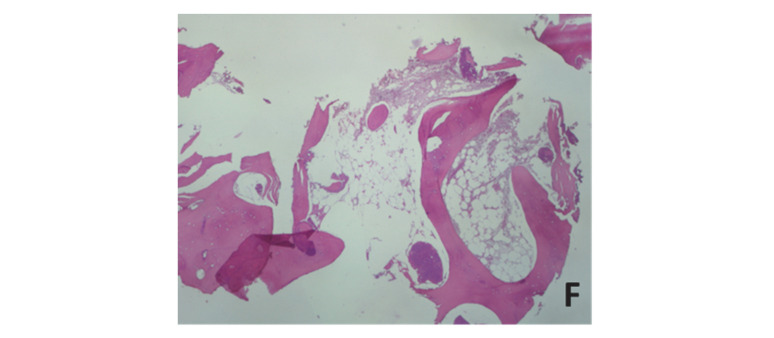
(**A**) Asymmetry of the lower third of the face; (**B**) Intense laterognathism of the mandible; (**C**) Wide mouth opening; (**D**) Panoramic X-ray showing the presence of osteoma of the left mandibular condyle; (**E**) 3D reconstruction showing the osteoma of the left condyle; (**F**) Mature trabecular bone with cellular adipose bone marrow H & E X40.

A 65-year-old male patient was presented to our clinic with a mild swelling of the left pre-auricular area, dating back over the last two years (Figure 3A). The swelling was hard in palpation, without pain. There was an obvious facial asymmetry and an occlusion disturbance (Figure 3B,C). Intra-oral examination revealed a cross-bite on the right side and with an interincisal distance of 35 mm during mouth opening. A temporomandibular joint examination was normal without audible sounds during mouth opening and his medical history was free from disease. No history of trauma or infection at the temporomandibular area was referred, and Gardner’s syndrome was also excluded taking into account medical history and past consultation.

Imaging of the affected area revealed a large peripheral osteoma of the left mandibular condyle consisting from compact bone and causing distortion of the lower third of the face (Figure 3D,E). Due to the size of the lesion, a biopsy through an extra-oral approach was decided on before surgical treatment. The biopsy confirmed the diagnosis of an osteoma of the left mandibular condyle (Figure 3F). The patient was informed of the details of the nature of the lesion and the reasons for surgical removal. However, he declined a surgical operation as the swelling was asymptomatic and had increased very slowly over the last years, and he was used to living with the existing disturbance of his jaw.

### 2.4. Case 4

A 42-year-old Caucasian female showed a painless left pre-auricular swelling that had been noticed 4 years before. A facial asymmetry was also observed by the patient 10 years ago, which had been gradually increasing since then. The patient was in overall good systemic health with a negative history of trauma or infection in the region and no signs of Gardner’s syndrome.

**Figure 4:**
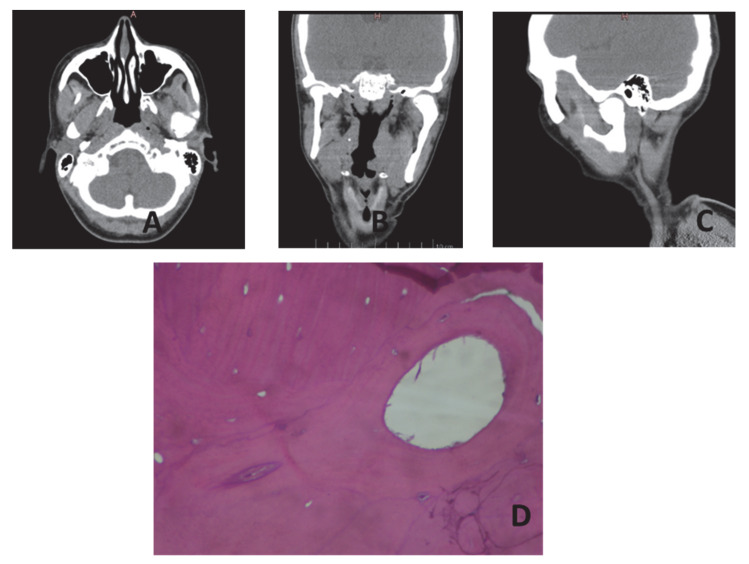
(**A**) Transverse section of CT imaging. There is an obvious enlargement of the left mandibular condyle; (**B**) Coronal view showing the affected left side in comparison with the healthy right side; (**C**) Oblique section of CT imaging. The enlarged left condyle is depicted; (**D**) Condylar osteoma consisting of osteocytes and haversian canals H & E X400.

An examination of the face revealed the presence of a hard painless pre-auricular swelling located at the left mandibular condyle and associated with an obvious mandibular asymmetry. The skin over the swelling was of a normal color and texture, with no signs of infection, ulcerations, or scars. An intra-oral examination showed a deviation of the mandible to the right, which was maintained during mouth opening, and a malocclusion with a posterior open bite on the affected left side. The mouth opening was normal, without any difficulty in chewing. A computerized tomography (CT) study of the face revealed a large well-defined, unilateral, mushroom-like osseous mass arising from the upper aspect of the left mandibular condyle giving rise to a marked facial asymmetry and a chin displacement to the right (Figure 4A–C). Based on the history, as well as the clinical and imaging findings along with biopsy to exclude other entities with similar findings, the diagnosis of a peripheral osteoma of the mandibular condyle was established (Figure 4D).

The lesion was scheduled for surgical removal under general anesthesia with an extra-oral approach but the patient denied the surgical treatment, preferring the periodic monitoring of the lesion, as the swelling increased very slowly over time and the patient was free of additional clinical symptoms.

## 3. Discussion

While the true etiology of osteoma remains unclear, its occurrence may be divided into syndromic and non-syndromic origins. A typical example of a syndromic origin osteoma is Gardner’s syndrome, which comprises multiple intestinal polyps, mesentery and skin fibromas, cutaneous sebaceous cysts, multiple supernumerary teeth, and craniomaxillofacial osteomas, with a predilection for membranous bones, such as the maxilla and mandible [4,6]. Sondergaard et al. [7] (1993) indicated that mandibular osteomas were also found in most patients with the hereditary dominant pre-malignant syndrome familial adenomatous polyposis, and concluded that mandibular osteomas are probably genetic markers for the development of sporadic colorectal carcinoma. Smrithi et al. [8] (2012) showed another interesting correlation of familial history or genetic transmission that may be taken into account for osteoma. According to these authors, although the probability of an osteoma occurring in a mother and a child is 0.0016%, the predisposition to osteoma formation in humans is possibly dominantly inherited and may be a recessive trait for vertical transmission in inbred strains of mice.

Osteomas of non-syndromic origin have several possible contributing factors in pathogenesis, which include trauma, reactive, neoplasmatic or developmental causes, metaplasia, surgery, irradiation, chronic infection, the alteration of calcium metabolism, and genetics [6,9,10,11]. It has been especially suggested by many investigators that an osteoma could be a reactive condition triggered by local trauma which could induce the onset of the lesion in more susceptible sites, such as the angle or lower border of the mandible. Furthermore, the proximity of osteomas to masseteric muscle attachments might induce, through muscle traction alone or in combination with a trauma, the initiation of the lesion [4,5,8,12].

There is an important variation in the literature concerning the reported percentage in distribution of these lesions at different parts of mandible and maxilla [3,5,9,10]. However, there is an agreement that most osteomas affect the mandible more frequently than the maxilla [13,14]. Craniofacial osteomas are rarely located on the facial bones, and in most of the cases, their location is into the paranasal sinuses. Peripheral osteomas are usually located at the mandibular body, whereas the condyloid process of the mandible is rarely affected. A review of the literature, as it is shown below, revealed only a few cases of osteomas located on the mandibular condyle, though each case is reportable [15,16,17,18,19,20,21,22,23,24,25,26,27,28,29,30,31,32] (Table 1).

Symptoms of an osteoma of the condyloid process depend on its exact location, and the most frequent symptoms included trismus [16,19,20,23,24,26,29,30,31,32], swelling [22,23,25,30,31], malocclusion [15,17,18,20,23,32], and asymmetry of the face [15,17,21,26,28,29]. Pain [19,28,31] was recorded in only a few cases and difficulty in swallowing [27] was observed in only one case of a large osteoma which developed from the internal surface of the mandibular condyle and had intra-oral swelling. As a general rule, when an osteoma is develops away from the articular surfaces, usually its presence does not interfere with occlusion, and the main complaint of the patient is the extra-oral or rarely intra-oral swelling at the site of the condylar area, as we have also noticed in one of our patients. Osteomas that interfere with the TMJ, shift the mandible towards the healthy side, producing facial asymmetry in the lower third of the face and malocclusion, as it was observed in three of our cases.

The clinical examination usually reveals a painless extra-oral swelling located at the mandibular condyle, and sometimes an intra-oral swelling when the lesion arises from the internal surface of the mandibular condyle. The swelling is painless and hard in palpation and increases slowly in size over the years. In some patients, facial asymmetry and malocclusion are dominant in clinical examination [15,16,17,18,29,32]. A routine radiographic examination usually confirms the clinical suspicion of an osteoma because the lesion appears as a radio-opaque mass in the condyloid process with well-defined borders. However, the key exam for its diagnosis is a computerized tomography (CT) which depicts the exact location and dimensions of the lesion, along with its anatomic relations with important adjacent anatomic structures, which guided the surgical plan [32].

In a differential diagnosis, the most important issue is the exclusion of osteochondroma, osteoma, osteoblastoma, or other malignant lesions that may appear in the mandibular condyle [33]. When the diagnosis of an osteoma is established, the next step is to exclude Gardner’s syndrome, as the syndrome is associated with peripheral and central (skull, ethmoid sinuses, mandible, and maxilla) osteomas, which often develop before polyps in the intestine. Therefore, maxillofacial osteomas may be a genetic marker for the development of colorectal carcinoma, and early diagnosis may be, in some cases, lifesaving [4,6,10,34]. None of our patients presented any clinical, imaging, or laboratory signs of Gardner’s syndrome.

The preferred surgical treatment of osteomas, located at the mandibular condyle, is a condylectomy followed by a simple surgical excision (Table 1). The selection of surgical approach depends on the exact location of the lesion and its dimensions. Access to the lesion is performed through either an intra-oral or a pre-auricular incision, and in specific cases, with a bicoronal approach [35]. Exophytic osteoma at the external surface of the condyloid process, and especially at the base of the condyle, are best treated through intra-oral access, using special retractors to gain access into this deep area of the mandible. Then, the removal of the osteoma, with chisels or burns, is a relatively easy procedure. This maneuver was used successfully in the first of our cases. However, when an osteoma interferes with the TMJ or if it arises from the internal surface of the condyloid process, the extra-oral approach through a conventional pre-auricular procedure is preferred, as it gives the opportunity of a wide surgical field and a better exposure of the lesion. This approach was applied in our second case, without any transient or permanent complication from the patient’s facial nerve. In addition, the removal of the osteoma restored the patient’s occlusion and so there was no need for any orthodontic or orthognathic secondary operation.

Since osteomas are due to a proliferation of either cortical or cancellous bone, there are histologic variants which include compact or ivory type, cancellous, trabecular or spongy type, and mixed type [4,36]. In our cases, histopathologic examination confirmed the clinical diagnosis of osteomas and revealed that the tumors consisted of lamellar bone only, without trabecular structures or bone marrow spaces.

The recurrence of osteomas after surgical excision is extremely rare, whereas there are no cases of a malignant transformation of osteomas in the literature [37,38], as we have observed in two of our patients who accepted the proposed surgical option.

## 4. Conclusions

Peripheral osteomas are benign lesions that are located rarely at the mandibular condyle. They have a slow growth rate, but their position usually causes functional and/or aesthetic disturbances. When they arise from the external surface of the mandible, the main symptom is related to aesthetic disturbances. In cases with an osteoma associated with the enlargement of the mandibular condyle, functional disturbances are observed. Surgical excision of the condylar osteoma is challenging, but can be performed usually with an extra-oral approach, while in selected cases, an intra-oral approach is an alternative option.

## Figures and Tables

**Table 1:** Reported cases of mandibular condyle osteomas with a description of their characteristics [(L-C): left condyle; (R-C): right condyle].

No	Authors/Year	Age/Gender	Location	Clinical Symptoms	Treatment
1	Chen et al., 2003 [15]	27/M		Asymmetry-Malocclusion	Condylectomy
2	Siar et al., 2004 [16]	32/F	L-C	Trismus	Condylectomy
3	Mancini et al., 2005 [17]	19/F	L-C	Asymmetry, Malocclusion	Condylectomy
4	Ortacoglu et al., 2005 [18]	22/M	R-C	Malocclusion	Condylectomy
5	Yonezu et al., 2007 [19]	50/M	L-C	Pain, trismus	Excision
6	Cogburn et al., 2008 [20]	55/M	L-C	Trismus, Malocclusion	Endoscopic excision
7	Bjornland et al., 2009 [21]	46/M		Swelling, Asymmetry	Excision
8	Chaurasia 2009 [22]	45/F		Swelling	None
9	Almeida, de Oliveira Filho 2011 [23]	45/M		Swelling, Trismus, Malocclusion	Excision
10	Misra et al., 2013 [24]	22/M		Trismus	Condylectomy
11	De Souza et al., 2015 [25]	41/F	L-C	Swelling	Excision
12	Rajshekar et al., 2015 [26]	35/M		Trismus, Asymmetry	Condylectomy
13	Motlagh et al.2015 [27]	78/M	L-C	Difficulty in swallowing	Excision
14	Zafar et al., 2016 [28]	60/M		Pain, Asymmetry	Excision
15	De Souza et al., 2017 [29]	67/F	R-C	Trismus, Asymmetry	Condylectomy
16	Heitz et al., 2018 [30]	49/F	R-C	Swelling, Trismus	Condylectomy
17	Valente et al., 2019 [31]	52/F	R-C	Pain, Trismus, Swelling	Condylectomy
18	Ostrofsky et al., 2019 [32]	60/M	R-C	Trismus, Malocclusion	Condylectomy

## References

[1] Kruger G. (1984). Textbook of Oral and Maxillofacial Surgery.

[2] Longo F., Califano L., De Maria G., Ciccarille R. (2001). Solitary osteoma of the mandibular ramus: Report of case. J. Oral Maxillofac. Surg..

[3] Kaplan I., Nicolaou Z., Hatuel D., Calderon S. (2008). Solitary central osteoma of the jaws: A diagnostic dilemma. Oral Surg. Oral Med. Oral Pathol. Oral Radiol. Endod..

[4] Deliverska E. (2016). Peripheral osteoma of mandible—A case report and analysis of literature. J. IMAB.

[5] Kaplan I., Calderon S., Buchner A. (1994). Peripheral osteoma of the mandible: A study of 10 new cases and analysis of the literature. J. Oral Maxillofac. Surg..

[6] Al-Yahya S.N.S.H., Hamizan A.K.W., Zainuddin N., Arshad A.I., Ismail F. (2015). Mastoid osteoma: Report of a rare case. Egypt. J. Ear Nose Throat Allied Sci..

[7] Sondergaard J.O., Rusmussen M.S., Videbak H., Bernstein I., Myrhoj T., Kristiansen K., Sommer P., Bulow S. (1993). Mandibular osteomas in sporadic colorectal carcinoma. A genetic marker. Scand. J. Gastroenterol..

[8] Smrithi D.V., Jayanthi K., Dayananda B.C. (2012). Solitary Peripheral Osteoma at a Curious Site with an Ambiguous Etiopathogenesis: A Case Report and Review of Literature. Int. J. Oral Maxillofac. Pathol..

[9] Sayan N.B., Ucok C., Karasu H.A., Gunhan O. (2002). Peripheral osteoma of the oral and maxillofacial region: A study of 35 new cases. J. Oral Maxillofac. Surg..

[10] Woldenberg Y., Nash M., Bodner L. (2005). Peripheral osteoma of the maxillofacial region. Diagnosis and management: A study of 14 cases. Med. Oral Pathol. Oral Cir. Bucal.

[11] Shanavas M., Chatra L., Shenai P., Veena K., Rao P., Prabhu R. (2013). Multiple Peripheral Osteomas of Forehead: Report of a Rare Case. Ann. Med. Health Sci. Res..

[12] Larrea-Oyarbide N., Valmaseda-Castellón E., Berini-Aytés L., Escoda C.G. (2008). Osteomas of the craniofacial region. Review of 106 cases. J. Oral Pathol. Med..

[13] Schneider L.C., Dolinsky H.B., Grodkesk J.E. (1980). Solitary peripheral osteoma of the jaws: Report of case and review of literature. J. Oral Surg..

[14] Dalabiras S., Boutsioukis C., Tilaveridis I. (2005). Peripheral osteoma of the maxilla: Report of an unusual case. Oral Surg. Oral Med. Oral Pathol. Oral Radiol. Endod..

[15] Chen Y.K., Lin L.M., Lin C.C., Hsue S.S., Huang E., Chuong S.Y., Chou S.H. (2003). Peripheral osteoma of the mandibular condyle. J. Chin. Med. Assoc..

[16] Siar C.H., Jalil A.A., Ram S., Ng K.H. (2004). Osteoma of the condyle as the cause of limited-mouth opening: A case report. J. Oral Sci. Mar..

[17] Mancini J., Woltmann M., Felix V., Freitas R. (2005). Peripheral osteoma of the mandibular condyle. Int. J. Oral Maxillofac. Surg..

[18] Ortakoğlu K., Gunaydin Y., Aydintug Y.S., Safali M. (2005). Osteoma of the mandibular condyle: Report of a case with 5-year follow-up. Mil. Med..

[19] Yonezu H., Wakoh M., Otonari T., Sano T., Hashimoto S., Uchiyama T. (2007). Osteoma of mandibular condyle as cause of acute pain and limited-mouth-opening: Case report. Bull. Tokyo Dent. Coll..

[20] Cogburn A.C., Hales N., Krempl G.A. (2008). Endoscopic resection of a mandibular condyle osteoma: Report of a case. Laryngoscope.

[21] Bjornland T., Berstad J.R., Store G. (2009). Peripheral osteoma of the mandible mimicking an ectopic condyle: A case report. Oral Surg..

[22] Chaurasia A., Balan A. (2008). Osteoid osteoma of jaws: An overview. J. Indian Acad. Oral Med. Radiol..

[23] Almeida L.E., de Oliveira F.M.A. (2011). Giant mandibular condyle osteoma. J. Craniofac. Surg..

[24] Misra N., Srivastava S., Bodade P., Rastogi V. (2013). Osteoma of temporomandibular joint: A rarity. BMJ Case Rep..

[25] De Souza P.D., Leonhardt F.D., Ahumada N.G., Abrahão M., Cervantes O. (2015). Giant osteoma of the mandible. Braz. J. Otorhinolaryngol..

[26] Rajshekar V.M., Basetty N.R., Govindaraju R., David M.P. (2015). “Out of the ordinary”: A case report of osteoma of mandibular condyle. J. Indian Acad. Oral Med. Radiol..

[27] Motlagh M.F., Janbaz Y., Abaszade A. (2015). Hyge peripheral osteoma of the mandibular condyle: A case report. J. Oral Maxillofac. Surg. Med. Pathol..

[28] Zafar E., Akbar Z., Niazi K., Pasha B. (2016). Osteoma of mandibular condyle-a case report. Pak. Oral Dental J..

[29] De Souza N.T., Cavalcante R.C.L., Cavalcante M.A.D.A., Hespanhol W., Jr. M.R.D.O., Ferreira D.D.C., Coutinho T.M.D.C., Gonçalves L.S. (2017). An unusual osteoma in the mandibular condyle and the successful replacement of the temporomandibular joint with a custom-made prosthesis: A case report. BMC Res. Notes.

[30] Heitz C., Conci R.A., Tomazi F.H.S., Louzada G.P., Guarenti M.M., Fritscher G.G. (2018). Giant Peripheral Temporomandibular Osteoma With Immediate Reconstruction of Mandible. J. Craniofac. Surg..

[31] Valente L., Tieghi R., Mandrioli S., Galiè M. (2019). Mandibular condyle osteoma. Ann. Maxillofac. Surg..

[32] Ostrofsky M., Morkel J.A., Titinchi F. (2019). Osteoma of the mandibular condyle: A rare case report and review of the literature. J. Stomatol. Oral Maxillofac. Surg..

[33] More C.B., Gupta S. (2013). Osteochondroma of mandibular condyle: A clinic-radiographic correlation. J. Nat. Sci. Biol. Med..

[34] Iatrou I.A., Leventis M.D., Dais P.E., Tosios K.I. (2007). Peripheral osteoma of the maxillary alveolar process. J. Craniofac. Surg..

[35] Norman J.E., Bramley P. (1990). A Textbook and Colour Atlas of the Temporomandibular Joint.

[36] Rodriguez Y., Baena R., Rizzo S., Fiandrino G., Lupi S., Galioto S. (2011). Mandibular traumatic peripheral osteoma: A case report. Oral Surg. Oral Med. Oral Pathol. Oral Radiol. Endod..

[37] Pereira C.U., de Carvalho R., de Almeida A., Danta R. (2009). Mastoid Osteoma. Consideration on Two Cases and Literature Review. Intl. Arch. Otorhinolaryngol..

[38] Singh R., Bhure S., Krishna B.P., Mazhar H., Thomas A., Soni S.K. (2020). Cancellous osteoma of temporomandibular joint. J. Dent. Res. Rev..

